# Investigating the Thermal Cracking Processes of a Concrete Disk Considering the Influences of Aggregates and Pores: A Numerical Study Based on DEM

**DOI:** 10.3390/ma19091759

**Published:** 2026-04-25

**Authors:** Song Hu, Xianzheng Zhu, Jian Shi, Yifei Li, Shuyang Yu

**Affiliations:** 1 School of Transportation and Civil Engineering, Nantong University, Nantong 226019, Chinay1287814ping@163.com (X.Z.); 2 Department of Mechanical Engineering, Huzhou University, Huzhou 313002, China

**Keywords:** concrete thermal cracking, discrete element method, microscopic model of concrete disk, deep geothermal environment, crack propagation law

## Abstract

In deep geothermal engineering, concrete slabs are prone to thermal cracking. The aggregates and pores are the core influencing factors for this failure behavior. However, existing research methods are unable to accurately capture the microscopic evolution process of thermal cracking and cannot clarify the intrinsic mechanism of how the characteristics of aggregates and pores affect the initiation and propagation of cracks. This limitation restricts the in-depth understanding of the laws of concrete thermal cracking. To address this deficiency, this study employs the discrete element method (DEM) and combines the particle flow program PFC2D to construct a microscopic model of concrete disks. By setting reasonable temperature parameters and thermal load boundaries, a numerical simulation system matching the actual deep geothermal high-temperature environment is established. Three sets of quantitative variables were designed: aggregate particle size (0.003, 0.004, 0.005, 0.006), aggregate volume fraction (0.35, 0.40, 0.45, 0.50), and porosity (0.11, 0.12, 0.13, 0.14). Through controlled variable simulations, the influence laws of each variable on the formation, propagation path, and time evolution of concrete thermal cracks were explored. The quantitative research results show that an increase in aggregate particle size significantly accelerates the generation and propagation of cracks. When the particle size is 0.006, the number of cracks is the highest and the propagation rate is the fastest. The aggregate volume fraction is negatively correlated with the final number of cracks, and 0.50 is the optimal fraction, at which the number of cracks is the smallest. A decrease in the fraction will lead to intensified stress concentration in the cement paste and a sudden increase in the number of cracks. An increase in porosity significantly disrupts the material continuity. When the porosity is 0.14, the bifurcation and connection of cracks are the most significant, while a low porosity of 0.11 can effectively inhibit the overall development process of thermal cracks. In addition, compared with traditional experimental methods and continuous medium numerical simulation techniques, the discrete element method has unique advantages in revealing the internal mechanism of concrete thermal cracking at the microscopic level. It can achieve real-time tracking of the evolution of discrete micro-cracks and the internal stress distribution characteristics. This study enriches the microscopic theoretical system of concrete thermal cracking and provides reliable quantitative references and technical support for the design of thermal crack resistance of concrete in deep geothermal engineering and the optimization of material composition.

## 1. Introduction

Under the guidance of the “dual carbon” goal, the development and utilization of geothermal energy as a clean and renewable energy source has received much attention. Deep geothermal engineering for geothermal development (such as dry hot rock extraction and construction of geothermal wells) has become an important direction in the energy strategic layout [1,2]. Concrete slabs, as core structural materials for geothermal well lining and waterway tunnel support, have long been in extreme environments with high temperatures, high stress, and water–heat–force coupling effects. The problem of thermal cracking has become increasingly prominent, seriously restricting the safety of the project and the efficiency of extraction [3,4,5]. The thermal cracking is mostly caused by high temperature gradients, thermal expansion constraints, hydration heat accumulation, and fluid seepage coupling, manifesting as crack expansion of the lining, peeling of the joint surface, and even structural collapse [6]. The development wells of dry hot rock in the Gonghe Basin of Qinghai Province once suffered from lining thermal cracking, resulting in the instability of the well wall and directly affecting the continuous operation of the geothermal extraction system. Therefore, conducting research on the mechanism of concrete thermal cracking, clarifying the laws of the influence of temperature on concrete cracking, has important theoretical and practical significance for geothermal development projects.

The research on concrete thermal cracking mainly includes three aspects: experimental, theoretical and numerical simulation. The experimental research mainly uses a high-temperature simulation test device to replicate the water–heat–force coupling conditions of the deep geothermal environment, and explores the mechanical performance evolution and crack initiation laws of concrete slabs under different geothermal gradients. The signal characteristics during the thermal cracking process are monitored in real time using acoustic emission technology, and the intrinsic mechanism of concrete thermal cracking is revealed by combining with microscopic characterization methods, such as: Jia et al. [7] used a customized test system, combining three-day and 28-day direct shear tests with scanning electron microscopy (SEM), X-ray diffraction (XRD), and computed tomography (CT) multi-scale characterization techniques. The study investigated the unilateral heating condition of tunnels in actual environments (temperatures of 50 °C and 95 °C). Peng et al. [8] used a new test system (MUGTCS) to study the spatial–temporal thermal damage effects of temperature and humidity gradients on concrete hydration reactions, microstructure and mechanical properties. Hu et al. [9] conducted a series of indoor accelerated exposure tests to systematically measure the quality loss, mechanical properties, microstructure evolution and ultrasonic wave propagation characteristics of concrete, and studied the damage mechanism of concrete under thermal-erosion coupling action. Li et al. [10] used uniaxial compression experiments to study the fracture and spalling phenomena around the main cracks under the influence of high-temperature thermal damage. He et al. [11] used experiments to construct a temperature gradient field to study the microscopic structural characteristics and mechanical properties of damaged concrete after damage. Zhang et al. [12] used a test device that couples the simulated temperature field with sulfate unidirectional erosion to measure the internal temperature, strength changes, damage layer thickness, pore structure and erosion product distribution of concrete, and studied the unidirectional erosion effect of concrete. Cui et al. [13] used mechanical tests to study the deterioration mechanism of high-temperature tunnels in a 100 °C dry heat environment. Zheng et al. [14] utilized the established on-site comprehensive multi-field information monitoring platform to study the interaction between the rock mass of the reservoir bank slope and the anchoring structure, as well as the long-term safety of the rock mass-anchoring structure system. Ma et al. [15] conducted on-site investigations and conducted triaxial shear tests on the original Q3 and Q2 loess samples with different moisture contents to study the deformation and failure characteristics and mechanisms of loess slopes induced by excavation in the Yan’an land reclamation project. They revealed the failure modes of loess slopes in different strata and provided support for the design and management of related projects. Tian et al. [16] conducted vibration test experiments using detailed geological surveys, historical seismic data, satellite optical images and unmanned aerial vehicle (UAV) aerial photography to study the multi-level seismic damage characteristics and progressive deformation process of the landslide in Xinmo Village, Sichuan Province. They revealed the deteriorating effect of historical seismic cumulative damage on the rock mass of the landslide and summarized its progressive deformation failure mechanism. Yang et al. [17] utilized the early plate crack resistance test to investigate the effects of silica fume, expansive agent, and crack-resistant waterproofing agent on the crack resistance and water permeability of concrete, optimized the mix ratio, and clarified the mechanism of their actions.

However, although the existing concrete thermal cracking experimental research can replicate the deep geothermal water–heat–force coupling environment through simulation devices and capture macroscopic cracking phenomena and signal characteristics, there are still obvious limitations. Most experiments can only explore the external cracking process and surface patterns, and it is difficult to intuitively display the real-time internal stress changes during the crack expansion and evolution process, and cannot fully restore the multi-scale coupling response of thermal cracking under complex deep conditions.

Theoretical research is based on experimental research, constructing a microscopic model of concrete thermal cracking, and quantitatively analyzing the coupling effect of temperature, stress and seepage. Combining the theory of fracture mechanics, the criterion for thermal crack propagation is derived to make up for the deficiency of experiments in characterizing internal stress and microscopic mechanisms. For example, Huang et al. [18] used a new unsaturated frozen soil thermal-water-hydraulic coupled model with an integrated volume strain correction term to study the effect of temperature-dependent volume strain changes in different soil layers on heat and water migration. Kuang et al. [19] used a crack-through Brazilian disk (CSTBD) numerical model applicable to layered sandstone to study the fracture characteristics of layered sandstone under splitting conditions after high-temperature treatment. Li et al. [20] used the thermal-water-hydraulic (THM) coupling initiation rate calculation formula for brittle rocks to study the relationship between the calculated and experimental values of crack initiation rate and temperature and water pressure. Wen et al. [21] used a new non-associated thermal–viscoplastic damage model to study the creep property of rocks. Zhang et al. [22] used the thermal-hydraulic coupled sub-ensemble Boltzmann–near-field dynamics (LB-PD) model to study the thermal cracking process at the microscopic scale of concrete. Zhang et al. [23] used a microscopic-scale multi-physics field computational model based on near-field dynamics to study the deterioration problem of reinforced concrete caused by chloride ion erosion. Li et al. [24] used a completely new microscopic-scale thermal–hydrodynamic coupling numerical model to study the full life cycle damage evolution law of concrete joints under the coupling effect of temperature and vehicle load. Deng et al. [25] utilized the piezoelectric active sensing method in conjunction with hybrid machine learning algorithms to fabricate laminated rubber bearings with different degrees of cracking damage and obtain detection signals. They then studied the detection effectiveness and application prospects of this method for assessing the degree of bearing cracking damage. Zhang et al. [26] established a theoretical model using Euler beams and the plastic hinge theory, designed a double-curved DREC negative Poisson’s ratio metamaterial, and conducted quasi-static compression tests to study the dual stress platform, multi-stage deformation characteristics, and mechanical behavior of this metamaterial.

However, existing theoretical research often requires simplifying assumptions for complex underground conditions, ignoring factors such as dynamic fluctuations of ground temperature, non-uniform distribution of seepage field and randomness of internal defects in concrete, resulting in deviations between theoretical results and engineering reality and making it difficult to accurately depict the multi-scale coupling evolution mechanism of thermal cracking.

Numerical simulation, as the third means of scientific research, can precisely replicate the process of concrete thermal cracking as long as a reasonable constitutive model and parameters are assigned. The existing numerical simulation methods are mainly divided into continuous methods and discontinuous methods. Among them, continuous methods include finite element method and finite difference method, which have the advantage of efficiently characterizing the distribution of deep macroscopic thermal–structural-seepage coupling fields and can quickly output the overall stress–strain response and thermal cracking trend of concrete, such as Dai et al. [27] who used the finite element simulation method to include both the tunnel excavation and geothermal operation stages in the analysis scope, and studied key rock mass parameters such as stiffness, permeability, stress ratio, and Biot coefficient, as well as site conditions, on the impact of thermal action on tunnel structure response and rock–soil response. Li et al. [28] used the finite element framework and the alternating Newton–Raphson iterative algorithm to solve the coupled control equations of displacement–temperature–moisture–degree–damage, and studied how to predict the cracking behavior of concrete in the early age. Cui et al. [29] used three-dimensional non-steady-state heat–water coupled finite element analysis to study the heat transfer performance of tunnel lining. Wang et al. [30] used the finite difference model to study the thermal-water-soluble coupling behavior of lining concrete under high-temperature action. Zou et al. [31] used the finite difference method to study the bonding–slip relationship of composite material–concrete interfaces. Sun et al. [32] used the finite element method to study the fire damage of concrete structures. Zhu et al. [33] used the finite element method to study the energy absorption capacity of concrete specimens under drop hammer impact and the entire impact process. In addition, the smoothed particle hydrodynamics method (SPH) overcomes the shortcomings of the grid method and can simulate large deformation problems, such as Yu et al. [34] who used the smoothed particle hydrodynamics method (SPH) to simulate the brittle failure process at the particle level by modifying the derivative terms of the traditional smoothed particle hydrodynamics kernel function, studying the degree of surrounding rock damage during the excavation process of the Jinping deep-buried tunnel. Yu et al. [35] used the smoothed particle hydrodynamics method (SPH) to study the internal mechanism of homogeneous and heterogeneous tooth cracking. Yu et al. [36] used the smoothed particle hydrodynamics method (SPH) to study the thermal-water-force-damage (THMD) of rock mass structure. Yu et al. [37] used the smoothed particle hydrodynamics (SPH) to study the water pressure fracturing process of sand–gravel rock strata. Yu et al. [38] used smoothed particle hydrodynamics (SPH) to study the fracture process of rock medium. Yu et al. [39] used the smoothed particle hydrodynamics (SPH) to study the discontinuity problem of the non-steady state of a sand–gravel dam under high water pressure during the water pressure fracturing process. Yu et al. [40] used the smoothed particle hydrodynamics (SPH) to study the cracking laws of grouting/ungrouting fissure tunnel specimens. Yu et al. [41] utilized the SPH framework in conjunction with the thermal-water-structural-damage (THMD) coupling model to investigate the expansion law of concrete cracking under the ice phase change in fissure water, as well as the influence of fractures, aggregates, and pores on the freezing cracking morphology.

However, the continuous methods have weak ability to characterize discrete micro-cracks in the concrete interior and are difficult to accurately capture the microscopic evolution process of crack initiation, bifurcation and penetration. It is based on the assumption of continuous medium, and the accuracy of characterizing the local damage concentration effect of concrete under deep conditions is insufficient, and there is a deviation from the actual microscopic fracture characteristics in engineering. Discontinuous methods include numerical manifold method, discrete element method, etc. The numerical manifold method has the advantages of both continuous medium and discrete medium simulation and can accurately characterize the entire evolution process of micro-cracks in concrete, such as initiation, bifurcation and penetration under deep conditions. It does not require pre-determined crack paths and can efficiently couple the effects of thermal, mechanical and permeation fields.

For instance, Yu et al. [42] utilized the three-dimensional numerical manifold method and applied it to the calculation of temperature stress in large-volume concrete structures, studying the thermal and mechanical response characteristics of complex large-volume concrete structures during the layered pouring construction process. Xu et al. [43] used the numerical manifold method to conduct predictive analysis on the crack expansion of concrete gravity dams. Kang et al. [44] employed the three-dimensional numerical manifold method, embedding crack surfaces at the physical centroid and crack tip, to achieve local tracking of the crack surface, and conducted numerical simulations on the crack initiation and expansion process of six typical type-I fracture mechanics models. Li et al. [45] utilized the three-dimensional numerical manifold method, selecting multiple benchmark examples related to rock mass movement for verification calculations, and studying the movement and instability problems of discrete rock mass systems in geotechnical engineering. Su et al. [46] utilized the three-dimensional numerical manifold method to study the three-dimensional crack propagation process in brittle rock materials. Wang et al. [47] utilized the three-dimensional numerical manifold method to study the rock thermal fracture problem. Liang et al. [48] simulated the crack initiation and expansion process within the rock mass under the action of the temperature field, derived the relevant system control equations, and studied the thermal–mechanical (TM) coupling problem of fractured rock masses.

However, the numerical manifold method has low computational efficiency when dealing with deep underground large-scale engineering models, and is prone to a significant increase in time consumption due to the increase in mesh complexity. It also lacks detailed characterization of heterogeneous components of concrete (such as aggregates and mortar interfaces). The discrete element method is adept at depicting the discrete characteristics of heterogeneous components of concrete and can accurately reproduce the microscopic damage and crack behavior of aggregates and mortar interfaces. It can efficiently simulate the large displacement and large deformation failure process of structures under complex underground conditions, without relying on continuum assumptions and has better adaptability, such as: Yu et al. [49] used the discrete element method (DEM) to study the weak interlayers in rock masses. Yu et al. [50] used the discrete element method (DEM) to study the interaction mechanism between holes and cracks under compressive shear loading (including the crack initiation mechanism induced by stress concentration and the influence laws of different crack morphologies on the interaction strength). Yu et al. [51] used the discrete element method (DEM) to study the fracture mechanism of layered rock masses. Yu et al. [52] used the discrete element method (DEM) to study the holes and cracks in rock masses. Yu et al. [53] used the discrete element method (DEM) to study the failure mechanism of intermittent cracks under compressive shear loading. Yu et al. [54] used the discrete element method (DEM) to study the fracture mechanism of V-shaped crack rock masses under compressive shear stress. Yu et al. [55] used the discrete element method (DEM) to study the fracture mechanics characteristics of S-shaped crack rock masses.

It is worth noting that although this study focuses on the thermal cracking problem of concrete in deep geothermal environments, the research on rock and soil engineering cracking also includes the freeze–thaw cracking of rocks in cold regions. For instance, Yu et al. [56] based on the mechanisms of rock freeze–thaw damage and water ice phase change cracking, studied the deterioration of defective rocks under the action of freeze–thaw cycles, providing a basis for disaster prevention in rock engineering in cold regions. Therefore, this paper adopts the discrete element method for numerical simulation.

Based on the shortcomings of previous studies, this paper uses the discrete element method to establish a micro-scale model of a concrete disk. Considering the effects of different concrete aggregate particle sizes, aggregate percentages, and porosity, it conducts a simulation of the thermal cracking process of the concrete disk, analyzes the crack propagation process of the concrete disk, and statistically studies the evolution law of cracks over time steps, thereby clarifying the thermal cracking mechanism of the concrete plate. The research results can provide a reference for the geothermal development in deep underground engineering.

## 2. The PFC Principle

### 2.1. The Basic Principles of PFC Temperature Simulation

**Figure 1:**
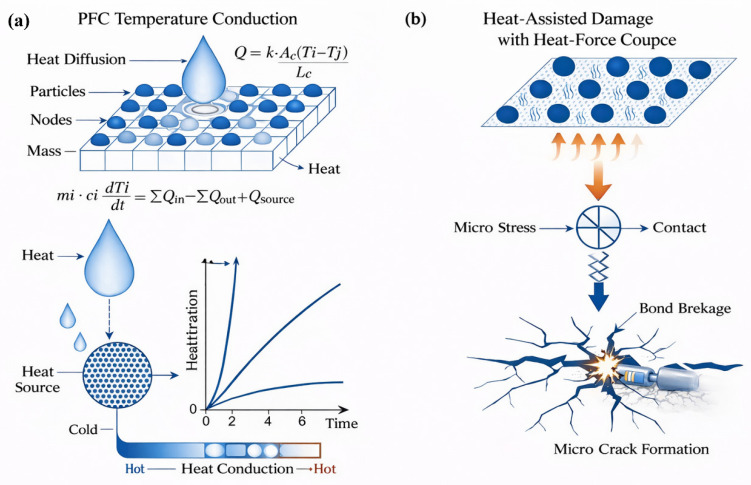
The thermal damage process of PFC particles.

In PFC, the temperature field is realized by introducing thermal degrees of freedom in the discrete particle system, as shown in Figure 1. The core idea is to consider the rock mass as a collection of a large number of particles, and couple the heat conduction and thermal expansion effects at the particle scale, thereby simulating the thermal–physical response behavior of the rock mass under temperature variations.

In the PFC thermal module, the thermal network approach is adopted. Each particle is a thermal node, with temperature (T_i), specific heat capacity (c_i), and mass (m_i). The contact between two particles constitutes a thermal conduction channel, and the heat flow Q satisfies:

(1) $$Q=k_{c}\cdot A_{c}\cdot \frac{T_{i}-T_{j}}{L_{c}}$$

Among them, k_c represents the contact thermal conductivity, A_c represents the effective heat transfer area of the contact, and L_c represents the length of the heat conduction path of the contact. The temperature within the particles is uniform, and the thermal inertia exists only in the particles, not at the contact. The evolution of the particle temperature usually follows a discrete form of the thermal equilibrium equation, and its essence is to achieve the transfer and distribution of heat within the system through contact heat transfer between the particles and boundary temperature conditions. When an initial temperature or fixed temperature boundary condition is applied to the particles, a stable or unstable temperature field will gradually form within the model. The energy balance of each particle is:

(2) $$m_{i}c_{i}\frac{dT_{i}}{dt}=\sum Q_{in}-\sum Q_{out}+Q_{source}$$

Among them, Q_in and Q_out represent the heat fluxes from the contact, while Q_source is the internal heat source. This implies that the change in particle temperature over time is determined by the net heat flux.

### 2.2. Temperature-Induced Fracture and Thermal-Force Coupled Fracture Mechanism in PFC

**Figure 2:**
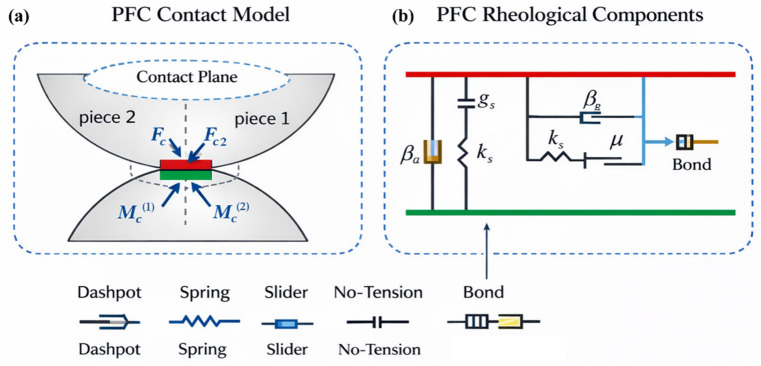
PFC particle contact. (**a**) Contact plane. (**b**) Rheological components.

The temperature-induced fracture in PFC is essentially a thermal–mechanical coupling failure process. The fracture mechanism does not directly generate cracks by temperature but is achieved through the redistribution of contact forces and bond breakage induced by thermal expansion, as shown in Figure 2, which presents the general particle contact model and the rheological mechanism between particles. When the particle temperature rises, the particles will expand in volume according to their thermal expansion coefficient. In a constrained system, the degree of thermal expansion of different regions of the particles is not consistent, thus generating significant thermal-induced additional stress at the particle contact points. This additional stress will be superimposed on the original mechanical stress, causing the local contact force to increase rapidly, especially at the tip of the pre-existing fracture, at the geometrically discontinuous areas, and in the regions with uneven particle arrangement.

In the numerical model, by gradually increasing the temperature, the thermal expansion effect accumulates over time and space, resulting in a significant temperature dependence in the initiation position, propagation direction, and propagation mode of the cracks. For specimens without cracks, high temperatures mainly induce random thermal damage, causing the specimen to transition from overall brittle failure to multi-crack co-operative failure; for specimens with cracks, the pre-existing cracks become the controlling structure for crack growth, and the crack initiation and propagation at the tip are more likely to occur. After heating, the particles generate thermal strain, thereby creating thermal stress at the contact points. Each particle has a thermal expansion coefficient α, and the target radius of the particles changes with temperature:

(3) $$R_{target}=R\left[1+\alpha \left(T-T_{0}\right)\right]$$

Under conditions of multiple fractures or fractures with different inclinations, the temperature-induced thermal stress in DEM alters the stress interaction mode between fractures, thereby influencing the deflection and bifurcation paths of cracks. This process is manifested macroscopically as a transition from a single crack controlling the failure mode to a collaborative evolution of multiple cracks. This fully reveals the amplification and modulation effects of temperature in the fracture process of fractured rock masses.

## 3. Numerical Model and Parameters

### 3.1. Carefully Reviewing Parameter Settings

In PFC, through the “init” and “fix” commands, initial temperature or constant temperature constraints are imposed on the particles within the specified range, thereby enabling the heating process of the samples within this range under high-temperature conditions. When the thermal module is activated, the change in particle temperature will directly affect their mechanical behavior. The temperature change will be converted into geometric deformation through the thermal expansion effect of the particles, and additional thermal strains and thermal stresses will be introduced at the particle contact points. In the PFC material settings, the parameters are defined as: fixed velocity displacement damping—damp, particle-specific heat capacity—sheat, particle thermal expansion coefficient—thexp, and particle initial temperature—temp.

Furthermore, in order to precisely simulate the thermal cracking behavior of the concrete disks in actual deep geothermal engineering scenarios, we also set the thermal load and temperature boundary parameters. These parameters were all determined based on the measured ground temperature fields in typical geothermal areas.

The initial temperature of the model is kept consistent with the surface construction environment temperature and the initial temperature of the particles in the microstructure parameter table. The target heating temperature is selected as the typical geothermal temperature range value of the deep geothermal well lining structure. In this concrete disk model, a uniformly distributed thermal loading boundary is adopted. The circular wall of the disk is set as the heat transfer boundary, and an insulation layer is set on the outside to eliminate external heat loss, ensuring that the temperature change in the internal particles is solely determined by the heat conduction between the particles and the preset thermal load.

The heating process adopts a linear heating rate, and the time step range from the initial temperature to the target temperature is completely matched with the time step interval for the subsequent crack evolution analysis, enabling synchronous monitoring of the accumulation of thermal load and the development of cracks. Throughout the heating process, the radial thermal gradient of the concrete disk is strictly controlled to be consistent with the actual thermal gradient distribution of the deep geothermal well concrete lining, ensuring that the temperature difference between the boundary and the center of the disk does not exceed the engineering practical range when reaching the target temperature. The above thermal load conditions are uniformly applied to the subsequent crack extension simulation and the time-step-based crack evolution analysis.

The various microscopic parameters of mortar and aggregates listed in Table 1 are all based on the actual test results. They are adjusted specifically according to the model characteristics of this study and the simulation conditions required for deep geothermal thermal cracking, and finally determined. It is also worth noting that when setting the parameters for Table 1, we referred to the research conducted by Zhu et al. [57] in this regard.

### 3.2. Computational Model and Dimensions

Concrete, as a typical multiphase composite material, has a microstructure mainly consisting of three components: mortar, aggregate, and pores. Each component has different compositions and performs distinct functions, collectively influencing the macroscopic properties of concrete.

**Figure 3:**
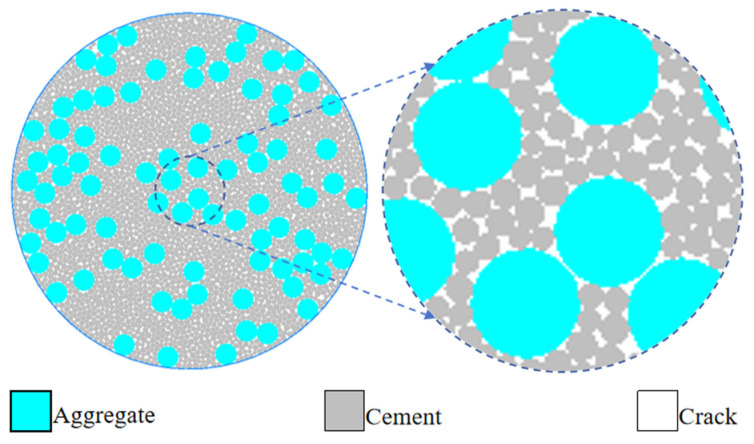
A microscopic model of the concrete disk.

In this paper, the PFC5.0 modeling software was utilized, and based on the modeling concept of PBM (Particle-scale Modeling), the generated model is shown in Figure 3. According to the physical and mechanical properties of concrete itself, the geometric parameters, particle size parameters of each phase, mechanical parameters, thermal parameters, and porosity parameters of the model were set. In terms of geometric parameters, the radius of the model was mainly defined, and a model size of 50 mm radius was selected. When setting the particle size parameters, the corresponding particle size intervals of aggregates, cement, and pores were set according to the grading of the selected concrete. When setting the mechanical parameters, the strength characteristics of each phase components and the physical laws of concrete cracking in practice were fully considered, and the elastic modulus and bond strength of aggregates and cement, as well as the bonding strength of pores, were set. When setting the thermal parameters, the thermal expansion coefficients of each phase components were mainly set. And when setting the porosity parameters, they were set in combination with the pore size of the concrete itself.

After the parameter definitions are completed, the construction of the model’s geometric boundaries begins. For the two-dimensional concrete disk model, the command for generating circular walls is used to construct the boundary of the disk. With the origin of the model coordinate system as the center and according to the preset radius, a complete circular wall is generated.

After the geometric boundary is constructed, the multi-phase particle generation based on PBM begins. Based on the selected concrete mix ratio characteristics, the volume fraction of the three-phase particles is set. In PFC, the generation of multi-phase particles is achieved through the particle distribution command. During the particle generation process, the area for particle generation is limited to the interior of the circular wall. After the particle generation is completed, the particles outside the disk boundary are cleaned, and all particles beyond the circular boundary range are deleted to avoid interference from the particles outside the boundary to the internal force and motion state of the model.

In addition, mechanical equilibrium processing was also carried out on the model to eliminate the initial stress generated during the particle generation process, ensuring that the model was in a stress-free state before thermal loading.

## 4. Crack Propagation Process

### 4.1. The Influence of Different Aggregate Particle Sizes on the Crack Propagation Process of Micro-Scale Concrete Plates

**Figure 4:**
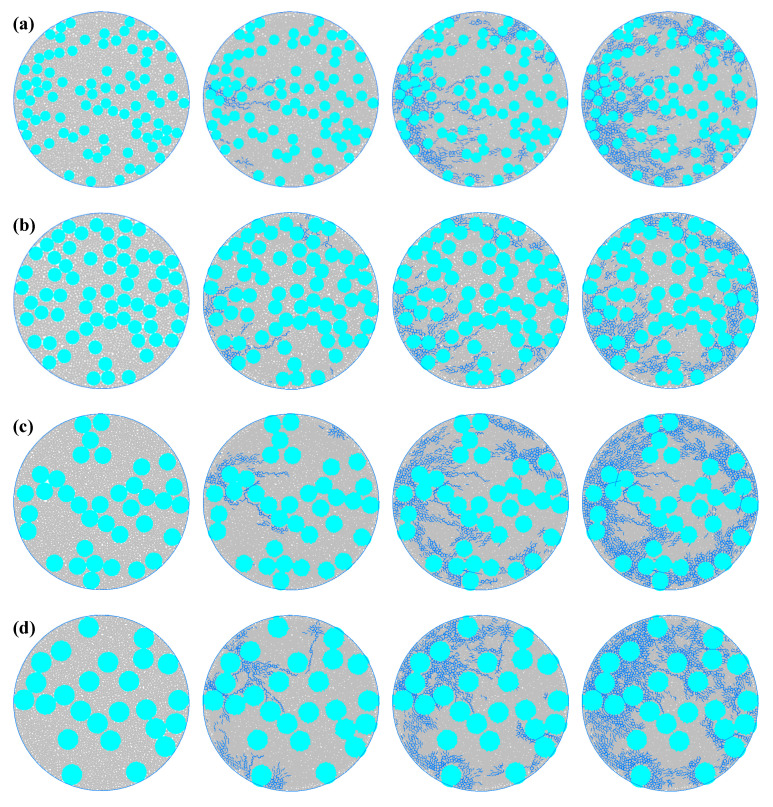
The failure process of the microstructure of concrete is shown under different aggregate particle sizes. (**a**) Aggregate particle size 0.003. (**b**) Aggregate particle size 0.004. (**c**) Aggregate particle size 0.005. (**d**) Aggregate particle size 0.006. (The blue one is the crack area).

Figure 4 shows the entire process of crack propagation in the concrete model under different aggregate particle sizes. Different aggregate particle sizes affect the density and toughness of the concrete, thereby influencing the crack initiation speed, initiation position, and propagation path. For group (a) (with the smallest aggregate particle size of 0.003), the aggregate particle size is relatively fine, and it can be more evenly and densely distributed within the concrete. The crack initiation and propagation process is relatively gentle. At the beginning of loading, the internal stress distribution is relatively uniform, and no obvious cracks are generated. In the middle of loading, a small number of cracks first appears at the edge area of the specimen. At this time, the cracks are characterized by a small number, short length, and a dispersed distribution. Some cracks begin to slowly extend towards the center and the loading directions above and below the specimen. Due to the relatively fine aggregate particles, the crack rarely exhibits a looping phenomenon, and no obvious bifurcated cracks occur during the extension process. In the later stage of loading, most cracks are still concentrated in the edge area of the concrete, and only a small number of cracks extend towards the central area, with limited extension length. For group (b) (with a slightly larger aggregate particle size than group (a), 0.004), the increase in aggregate particle size slightly reduces the uniformity of the aggregate distribution within the concrete. During the entire crack development process, the number of cracks and the extension speed slightly increase. In the middle stage of loading, more cracks extend towards the center and the up–down directions of the specimen. Due to the increase in aggregate particle size, the crack looping phenomenon is less frequent, the looping path is shorter, and no obvious bifurcated cracks occur during the extension process. In the later stage of loading, the number of cracks extending towards the center area increases. For group (c) (with an even larger aggregate particle size of 0.005), the further increase in aggregate particle size makes the uniformity of the aggregate distribution within the concrete more obvious, and the total number of cracks is greater than that of group (b), with a wider extension range. In the middle stage of loading, more cracks extend towards the center of the specimen. Due to the further increase in aggregate particle size, the crack looping phenomenon is more frequent, the looping path is shorter, and fewer bifurcated cracks occur during the extension process. In the later stage of loading, the cracks distributed in the edge area of the specimen increase in density, almost filling the entire edge area. For group (d) (with the largest aggregate particle size set at 0.006), as the largest aggregate particle size among the four groups of experiments, the distribution of concrete aggregate is the most uneven, and the total number of cracks is the largest among the four experiments, with the fastest extension rate. In the middle stage of loading, many cracks extended towards the center and the up-down directions of the specimen. During the extension process, the looping phenomenon is very common, and many bifurcated cracks are generated, and secondary bifurcated cracks also continuously occur. In the later stage of loading, the cracks occupy most of the area of the concrete plate.

### 4.2. The Influence of Different Ratios of Aggregates on the Crack Propagation Process of the Microstructure of Concrete Plates

**Figure 5:**
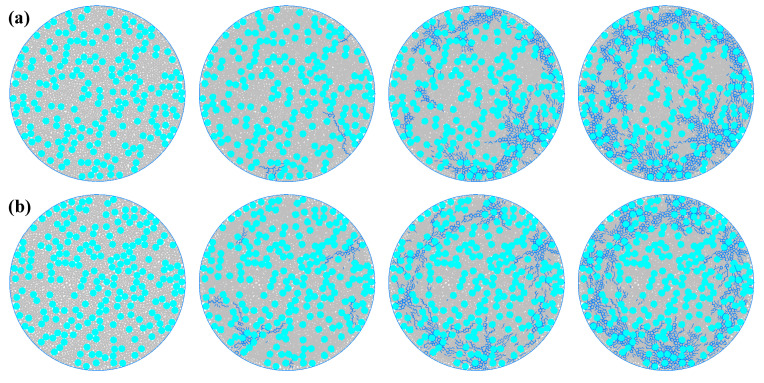

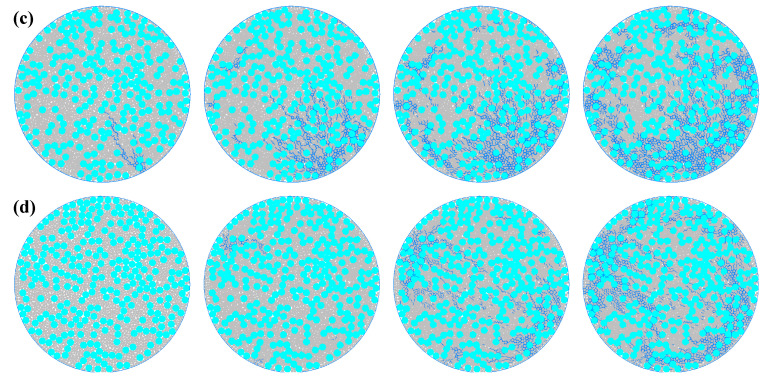
The failure process of micro-scale concrete under different aggregate ratios. (**a**) Aggregate ratio of 0.35. (**b**) Aggregate ratio of 0.40. (**c**) Aggregate ratio of 0.45. (**d**) Aggregate ratio of 0.50. (The blue one is the crack area).

Figure 5 shows the entire process of crack propagation in the concrete model under different aggregate ratios. Different aggregate ratios affect the number and mode of crack development by altering the homogeneity and mechanical properties of the concrete. For group (a) (with the smallest aggregate ratio of 0.35), the content of aggregates is low, unable to provide sufficient resistance when cracks develop in the concrete. The crack generation speed is fast and the number is large. At the initial loading stage, a few cracks appear at the edge of the specimen. In the middle loading stage, the cracks originally at the edge of the specimen extend to the central area. Due to the low aggregate content, the circling phenomenon of cracks lessens, and the bifurcated cracks during the extension process are also rare. In the later loading stage, the cracks further develop towards the center, forming a large area of crack zone. For group (b) (with a slightly higher aggregate ratio than group (a), 0.4), the increase in aggregate ratio leads to a larger distribution of large-grained aggregates in the concrete, blocking the expansion paths of a few cracks. The total number of cracks decreases. In the middle loading stage, compared to group (a), due to the increase in aggregate ratio, more circling phenomena of cracks occur, and more bifurcated cracks accompany the extension process. In the later loading stage, the number of cracks extending from the edge area to the center decreases. For group (c) (with an even higher aggregate ratio of 0.45), the further increase in aggregate ratio makes the aggregation of aggregates in the concrete more obvious, and the resistance to crack development also increases. The total number of cracks is smaller than that of group (b). In the middle and later loading stages, the number of cracks extending from the edge area to the center decreases, and they concentrate on extending towards the center. The further increase in aggregate ratio also leads to more circling phenomena of cracks during the extension process, and more bifurcated cracks. For group (d) (with the largest aggregate ratio of 0.5), as the group with the largest aggregate ratio among the four groups, its concrete aggregate content is the highest, and the total number of cracks is the lowest among the four experiments. Before and during the loading, only a few cracks appear at the edge of the specimen and extend towards the center. In the later loading stage, the number of cracks is also significantly smaller than the previous groups.

### 4.3. The Influence of Different Porosities on the Crack Propagation Process of Micro-Scale Concrete Plates

**Figure 6:**
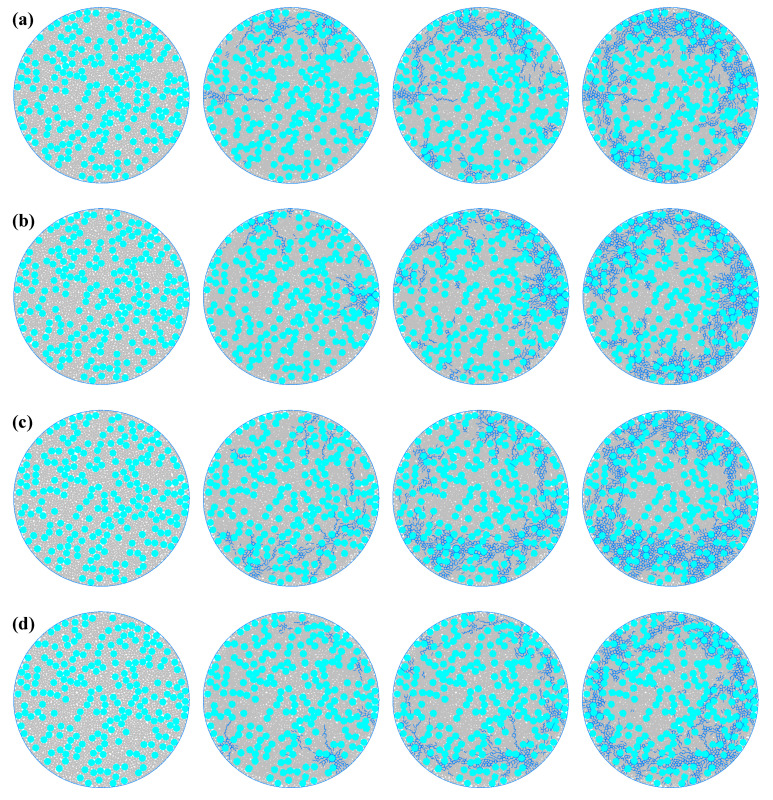
Microscopic concrete failure process under different porosities. (**a**) Porosity 0.11. (**b**) Porosity 0.12. (**c**) Porosity 0.13. (**d**) Porosity 0.14. (The blue one is the crack area).

Figure 6 shows the entire process of crack propagation in the concrete model under different porosities. Different porosities affect the propagation speed and distribution of cracks by altering the stress distribution of the concrete and the continuity of the materials. For group (a) (with the lowest porosity of 0.11), due to the low porosity and strong material continuity, there are fewer internal defects, which to some extent inhibits the propagation of cracks. The number of cracks is relatively small. At the beginning of loading, cracks start to appear slightly at the edges of the specimen. In the middle of loading, cracks at the edges of the specimen extend towards the central area, with fewer looping phenomena and fewer bifurcated cracks during the extension process. In the later stage of loading, cracks extend further towards the center, but mainly concentrate at the edge area, and the number is relatively small. For group (b) (with a porosity slightly larger than group (a), at 0.12), the porosity increases, and the number of pores also increases. The crack propagation speed increases. In the middle of loading, compared to group (a), there are more looping phenomena of cracks, and more bifurcated cracks occur during the extension process. In the later stage of loading, the number of cracks extending towards the center from the edge area increases. For group (c) (with a further increase in porosity to 0.13), the continuous increase in porosity enlarges the internal defects of the concrete, promoting the propagation of cracks. The total number of cracks is greater than that of group (b). During the middle and later stages of loading, the number of edge cracks increases, and they mainly extend towards the center. There are more looping phenomena of cracks during the extension process, and more bifurcated cracks occur, making the propagation path more tortuous. For group (d) (with the highest porosity of 0.14), as the group with the largest porosity among the four groups, the continuity is the worst, and the total number of cracks is the largest among the four groups. Before, during, and after loading, the number of cracks increases, the looping phenomenon becomes more obvious, and the number of bifurcated cracks increases, making the propagation path more tortuous.

## 5. The Variation Pattern of Cracks over Time Steps

**Figure 7:**
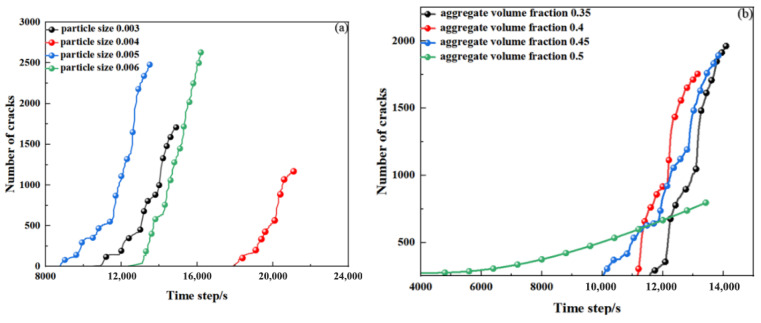

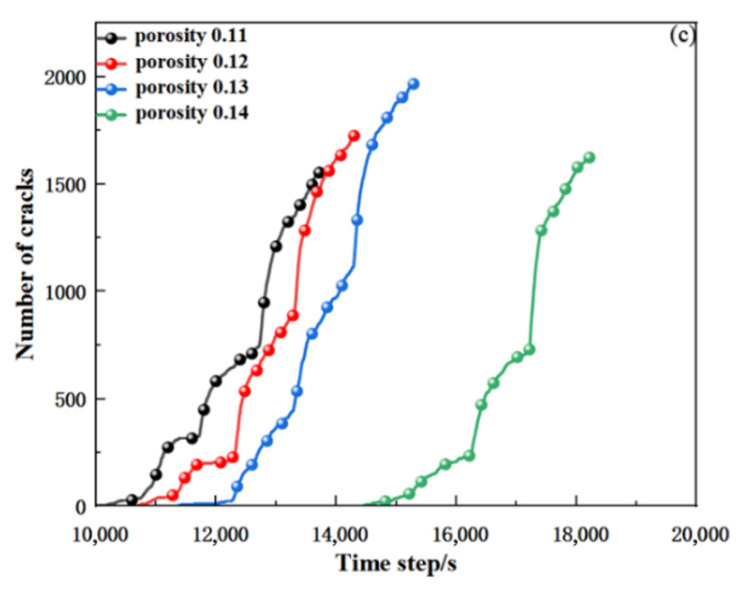
Number of microcracks in the interior of different concrete samples. (**a**) Different aggregate particle sizes. (**b**) Different aggregate proportions. (**c**) Different porosities.

As shown in Figure 7, for group (a) (with different aggregate particle sizes), the focus was on exploring the pattern of changes in the number of concrete cracks as the aggregate particle size increased. Under the condition that the simulated concrete was the same, cracks first appeared when the aggregate particle size was 0.005, followed by 0.003, then 0.006, and finally 0.004. Moreover, the time when cracks began to appear when the aggregate particle size was 0.004 was significantly later than the other three cases. Thus, it can be seen that an increase in aggregate particle size promotes the early occurrence of cracks. It is also notable that, in the case of smaller aggregate particle sizes, such as a particle size of 0.003, the time when cracks began to develop was earlier. Therefore, a too small aggregate particle size can also promote the early occurrence of cracks. By observing the slopes of the four curves and their trends, it can be found that the slope at the aggregate particle size of 0.006 is significantly greater than the other three curves. Thus, an increase in aggregate particle size promotes the speed of crack development at all stages. Additionally, from the final number of cracks, the number was the highest when the aggregate particle size was 0.006, which also indicates that an increase in aggregate particle size promotes the development of cracks to a certain extent, which is consistent with the conclusion obtained from the analysis of PFC images in the previous section.

For group (b) (with different amounts of aggregate), the focus was on exploring the pattern of changes in the number of concrete cracks as the proportion of aggregate increased. From the graph, the following situations can be observed: The cracks first appeared at an aggregate proportion of 0.5, and the slope was significantly smaller than the other three curves. The entire curve had an upward concave shape, with a long duration of crossing, and ultimately the number of cracks was the lowest. Next was an aggregate proportion of 0.45, with a smaller slope, a shorter crossing time, and ultimately a lower number of cracks. Then came an aggregate proportion of 0.4, with a larger slope, a shorter crossing time, and the number of cracks was similar to that at an aggregate proportion of 0.45. Finally, an aggregate proportion of 0.35 had the largest slope and a shorter crossing time, and ultimately the number of cracks was the highest. Thus, it can be seen that a reduction in the proportion of aggregate will to some extent promote the occurrence of cracks, which is consistent with the conclusion obtained from the analysis of PFC images mentioned earlier.

For group (c) (with different porosities), the focus was on exploring the pattern of changes in the number of concrete cracks as the porosity continuously increased. From the graph, the following situations can be observed: The porosity of 0.11 was the first to develop cracks, with the smallest slope, and the final number of cracks was the smallest. Next, the porosity of 0.12 developed cracks, with a smaller slope, and the final number of cracks was greater. Then, the porosity of 0.13 developed cracks, with a larger slope, and the final number of cracks was the largest. Finally, the porosity of 0.14 developed cracks, with the largest slope, and the final number of cracks was similar to that of 0.12. Thus, it can be seen that an increase in porosity will to some extent promote the subsequent development of cracks, but it will also prolong the initial appearance time of cracks. Moreover, it is worth noting that an excessively high porosity will to some extent reduce the final number of cracks. The overall trend of change is consistent with the conclusion obtained from the analysis of the PFC images mentioned earlier.

## 6. Quantitative Analysis of the Evolution Characteristics of Cracks

To improve the quantitative analysis system of the research, this study systematically analyzed core indicators such as crack density, main crack proportion, stress concentration coefficient, and fracture energy for concrete circular disk models with different aggregate particle sizes, mixing ratios, and porosities. It also fitted evolution parameters such as the crack initiation time step and the crack growth rate coefficient, and analyzed the coupling relationship between stress field evolution and crack development. This enabled the combination of qualitative crack characteristics and quantitative laws.

The crack density (total number of cracks per unit area) and the steady-state crack density significantly increase with the increase in aggregate particle size, the decrease in admixture ratio, and the increase in porosity. The increase in aggregate particle size promotes the penetration of micro-cracks to form main cracks, and the proportion of main cracks increases. Reasonably increasing the aggregate admixture ratio and reducing the porosity can effectively inhibit the development of main cracks. The interface between the aggregate and the mortar, as well as the surroundings of the pores, are the core areas of stress concentration. The larger the aggregate particle size and the higher the porosity, the greater the stress concentration coefficient and the amplitude of stress fluctuation. Increasing the aggregate admixture ratio can optimize the uniformity of the stress field and reduce stress concentration. Moreover, the time step of crack initiation and expansion is highly consistent with the time step of stress field mutation, and the expansion direction is consistent with the principal stress direction. The evolution of the thermal stress field is the core factor controlling the thermal cracking of concrete.

When the aggregate content is within a reasonable range, the fracture energy at the aggregate–mortar interface reaches its peak, and more energy is required for crack propagation. Excessively large aggregate particle size and increased porosity reduce the overall fracture energy of concrete, resulting in a significant decrease in energy dissipation during crack propagation. The crack propagation rate exhibits the same trend as the crack growth rate coefficient. Both are positively correlated with aggregate particle size and porosity, and negatively correlated with aggregate content. Either excessively large or small aggregate particle size, as well as increased porosity, advance crack initiation, while increasing aggregate content can effectively delay it. The higher the porosity, the larger the fractal dimension of cracks, the more tortuous the crack propagation path, and the more significant the branching and coalescence phenomena.

The above quantitative indicators are mutually consistent and highly consistent with the qualitative crack patterns. Among them, crack density, the proportion of main cracks, and stress concentration coefficient can be regarded as the core indicators for characterizing the degree of concrete thermal cracking. Fracture energy and crack propagation rate can be regarded as the key indicators for evaluating its thermal cracking resistance. This comprehensively reveals the internal mechanism by which aggregates and pores, through changing the uniformity of the microscopic structure of concrete, regulate the stress field and energy dissipation characteristics, ultimately influencing the initiation, expansion and connection of thermal cracks.

## 7. Discussion

### 7.1. The Coupling Effect of Aggregate Particle Size, Aggregate Content and Porosity on the Propagation of Thermal Cracks in Concrete

This study established a mesoscopic model of concrete disks based on the discrete element method and simulated the thermal cracking process under deep geothermal conditions. It interpreted the intrinsic mechanism by which aggregate particle size, dosage and porosity affect the initiation and propagation of thermal cracks from the essence of mesoscopic structure and stress transmission, and also clarified the coupling law of mutual regulation among the three.

The influence of aggregate particle size on thermal cracking essentially lies in altering the uniformity of the concrete microstructure and the bearing capacity characteristics of the aggregate–sand interface. When the particle size is too high, the uniformity of aggregate distribution decreases, and the interface bonding area reduces, forming a mechanically weak zone. The additional thermal expansion stress is prone to concentrate here, promoting the initiation, branching, and rapid expansion of cracks, which is consistent with the result in this study’s simulation where the increase in particle size leads to an increase in the crack propagation rate. Very fine aggregates will cause a significant increase in the proportion of cement mortar, and the concrete loses its main load-bearing framework of aggregates. The stress is mostly borne by the mortar with a lower tensile strength, causing local stress concentration and accelerating the initiation of cracks.

The core function of the aggregate content is to construct the internal load-bearing framework. Its influence is directly related to the integrity of the framework and the degree of gap filling. When the dosage is appropriate, the aggregate forms a continuous and stable main load-bearing framework. The cement mortar fully fills the gaps and eliminates the original defects, and the thermal stress is uniformly transmitted through the framework. This is the fundamental reason why the number of final cracks is the smallest under this condition in the simulation; when the dosage is too low, an effective framework cannot be formed, and the stress is mainly borne by the mortar, which is prone to cracking; when the dosage is too high, the mortar is difficult to fill the gaps of the aggregate, and a large number of pores is formed, becoming a new source of defects, destroying the integrity of the framework and causing stress concentration around the pores, which instead aggravates thermal cracking, which is consistent with the result that the number of cracks increases in the high-dosage group in the simulation. In summary, the particle size of aggregates, the proportion of aggregates, and the porosity all mainly affect the degree of tight arrangement of aggregates and cement, thereby influencing the situation of stress concentration within the concrete and the number of final cracks.

Porosity, as the core indicator of internal defects in concrete, regulates the development of thermal cracks by altering the continuity of materials and the effective load-bearing area. This aligns with the results of our simulation study, where the porosity significantly influences the branching and penetration of cracks. At low porosity levels, the material has strong continuity and uniform stress distribution, making crack propagation more difficult and requiring greater resistance, effectively inhibiting crack development. As the porosity increases, the material continuity is disrupted, and the stress concentration areas around the pores overlap, accelerating the branching and penetration of cracks. When the porosity exceeds the critical value, the internal pores are dispersed, and the stress concentration areas are difficult to connect, preventing the formation of macroscopic cracks from microcracks. Eventually, the number of cracks decreases, which also explains the nonlinear change characteristics of porosity and the number of cracks in the simulation.

The coupling effect of the three factors also relies on the microstructure of concrete and the stress transmission laws: The complete load-bearing framework formed by an appropriate amount of aggregate can effectively counteract the problem of material continuity decline caused by medium porosity; fine aggregates can fill the gaps in the framework of low aggregate content, improving the structural uniformity, optimizing the thermal stress transmission path, and alleviating the stress concentration of cement mortar under low aggregate content. This coupling law provides a microscopic optimization idea for the concrete mix ratio design of deep geothermal engineering, that is, through the coordinated regulation of multiple parameters, to construct a microscopic structure with uniform structure, complete framework, and controllable defects.

### 7.2. Advantages and Innovation Points of Discrete Element Method in the Study of Thermal Cracking of Concrete Disks

In this study, the discrete element method (PFC2D) was employed to simulate the thermal cracking of concrete disks. By leveraging the particle-scale analysis advantages of this method and the PFC thermal–structural coupling simulation principle, it overcame the limitations of traditional experiments and other numerical methods. Moreover, it revealed the intrinsic mechanism of thermal cracking at the microscale, closely aligning with the research characteristics of heterogeneous composite materials of concrete.

The core advantage of the discrete element method lies in its ability to reproduce the three-phase microstructural heterogeneity of concrete aggregates, mortars, and pores. It can assign independent mechanical and thermal parameters to each phase, accurately simulate the thermal expansion, contact adhesion, and fracture behaviors of each phase, and combine the PFC temperature simulation principle to achieve particle-level heat conduction and thermal stress coupling. It can also track the bonding and fracture processes between particles in real time, clearly depicting the entire process of thermal cracks emerging from the aggregate–mortar interface, around the pores, and expanding, bifurcating, and penetrating. This is something that traditional macroscopic experiments that only capture surface macroscopic cracks cannot achieve. At the same time, this method does not rely on the assumption of a continuous medium and can precisely characterize internal discrete micro-cracks, compensating for the shortcomings of finite element and other continuous medium methods in depicting discrete cracks, and also solving the problem of rough characterization of heterogeneous components by the smooth particle fluid dynamics method, making the simulation results more in line with the actual thermal cracking characteristics of concrete.

Furthermore, the parameter adjustability and simulation efficiency of the discrete element method perfectly meet the multi-condition comparison requirements of this study, which involve three sets of variables: particle size of aggregates, dosage, and porosity. By simply adjusting the model parameters, different conditions can be simulated quickly. Compared to traditional physical experiments, this significantly reduces cost and time. It also enables a more systematic exploration of the influence patterns of each variable. This transformation of the discrete element method from a purely numerical simulation to a research tool for interpreting engineering practical problems is also an important innovation point of its application in this study.

The application of the discrete element method in this study has demonstrated its unique value in revealing the microscopic mechanism of concrete thermal cracking at the particle scale, which compensates for the deficiency of traditional research that “emphasizes the macroscopic aspect while neglecting the microscopic aspect”. In the future, by integrating the water–heat–force coupling characteristics of deep geothermal engineering, and strengthening the multi-field coupling simulation capability of this method, it will be able to more accurately simulate the actual concrete thermal cracking process in engineering, providing more practical theoretical support for engineering design.

### 7.3. Validations of the Simulation Results

Furthermore, by comparing the PFC simulation results of our study with the microscopic experimental analysis results of Gao et al. [58], we found that the two sets of results were highly consistent in their core conclusions, thereby verifying the reliability of our simulation. Specifically, the experimental results of Gao et al. confirmed the key conclusions drawn in our study in the following three aspects.

Firstly, our conclusions regarding the particle size of aggregates and the composition of aggregates for crack initiation and propagation have been supported by the experimental results of Gao et al. For instance: in our simulation, the larger the aggregate particle size, the worse the uniformity of particle packing, and the more severe the incoordination of thermal expansion deformation, resulting in a significant local stress concentration around the aggregates, thereby lowering the threshold for crack initiation and accelerating its propagation, while Gao et al. [58] found that when the aggregate gradation contains overly coarse particles, the total porosity inside the concrete significantly increases, and the proportion of large-pore structures also increases. These overly coarse particles introduce more internal defects (such as large pores), and these defects are the structural roots for the preferential initiation and rapid expansion of thermal cracks, leading to faster crack propagation.

**Figure 8:**
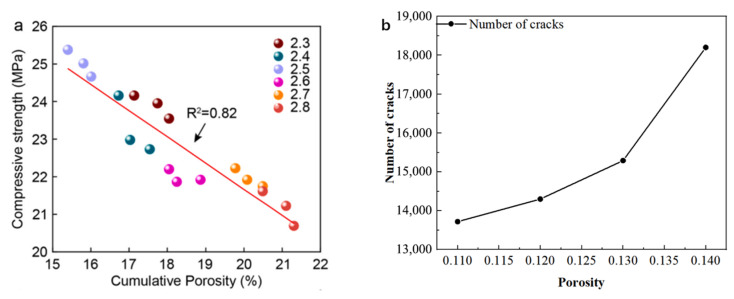
(**a**) The linear relationship and corresponding fitting goodness between compressive strength and porosity [58]. (**b**) The variation in the number of cracks under different porosities.

Secondly, regarding the correlation mechanism between porosity and mechanical properties, the simulation results show a consistent trend with the experimental results. This study found that, as shown in Figure 8, as the porosity increases, the internal defects in the material also increase, resulting in more severe stress concentration, which accelerates the initiation and propagation of cracks. This phenomenon has been explained in the experimental results of others. Their research indicates that the optimal gradation of D = 2.5 reduces the porosity of the ITZ area by 25.5%, thereby significantly improving the compressive strength. This directly supports the core viewpoint of this study: porosity is a key parameter determining the thermal crack sensitivity of concrete. Reducing porosity can effectively inhibit crack propagation and, to a certain extent, increase the strength of concrete.

Finally, in terms of the evolution mechanism of micro-defects, both studies revealed the same failure mode. This research observed that thermal cracks tend to generate stress concentration and bifurcate around the pores, while Gao et al., using deep learning (CNN) and metal intrusion method to extract micro-characteristics, indicated that deteriorated ITZ often presents a highly connected large-pore structure, while the optimized ITZ shows a dense spider-web-like structure. This difference in microstructure is completely unified with the mechanism of “cracks flowing around and bifurcating at the pores” simulated in this study.

## 8. Conclusions

(1) The size of the aggregate particles has a significant impact on the thermal cracking of concrete disks. The number of cracks and the rate of crack expansion generally increase with the increase in particle size. Specifically, when the aggregate particle size is small, the final number of cracks is smaller, and the phenomena of looping and branching during the crack expansion process are not obvious. This is because at this time, the specific surface area of the particles is large, they are more closely arranged, leaving fewer voids, which can inhibit the further generation of cracks, and the internal structure of the concrete is uniform, avoiding the generation of local stress.

(2) The proportion of aggregates affects the compactness of the particle arrangement within the concrete. As the proportion of aggregates increases, the number of cracks and their expansion rate decrease. When the proportion of aggregates is high, most of the cracks are concentrated at the edges, and a small portion extend towards the center, with a low crack development rate. This is because the increase in the proportion of aggregates enhances the role of aggregates as the main force-bearing component, thereby improving the bearing capacity of the concrete. The reduction in voids leads to a decrease in the number of inherent cracks within the concrete, resulting in a lower final number of cracks.

(3) Porosity, as a key parameter of internal defects in concrete, directly determines the material’s continuity and effective bearing area, thereby influencing the thermal cracking process. The number and expansion rate of cracks increase with the increase in porosity. Under low porosity conditions, the solid phase in the concrete accounts for a high proportion, and the stress distribution is uniform. Crack propagation requires overcoming greater resistance, so the number of cracks is small and concentrated in the edge areas. As the porosity increases, the material’s continuity is disrupted, the effective bearing area decreases, and stress concentration areas are easily formed around the pores, accelerating crack generation, branching, and penetration, ultimately leading to a more severe overall thermal cracking damage of the concrete.

(4) The Discrete Element Method (DEM) demonstrates significant advantages and reliability in the microscopic simulation of concrete thermal cracking. This method can overcome the observational limitations of traditional experiments and present the results with particle-level accuracy. During the process of concrete thermal cracking, it can clearly depict the entire process of crack initiation and expansion, as well as the microscopic behaviors such as the failure of the aggregate–sand mixture interface and the deflection of pore-induced cracks. Moreover, the Discrete Element Method can flexibly adjust key parameters such as the particle size, the proportion of aggregates, and the porosity, enabling rapid comparative analysis of multiple working conditions and providing an efficient tool for revealing the influence of various factors on thermal cracking. The research results provide theoretical support for the design of concrete for deep geothermal engineering to resist thermal cracking, and are of great significance for ensuring the safety and stability of geothermal development projects.

## Figures and Tables

**Table 1:** Microscopic parameters of PFC [57].

Parameters of Mortar		Parameters of Aggregates	
Emod (Pa)	130 × 10^8^	Emod (Pa)	555 × 10^8^
Pb_emod (Pa)	130 × 10^8^	Pb_emod (Pa)	555 × 10^8^
Pb_ten (Pa)	5 × 10^6^	Pb_ten (Pa)	20 × 10^6^
Pb_coh (Pa)	6 × 10^6^	Pb_coh (Pa)	25 × 10^6^
Pb_PFA (°)	45	Pb_PFA (°)	40
Kratio	2	Kratio	1.5
damp	0	damp	0
Temp (K)	283	Temp (K)	283
Sheat (J/(kg⋅K))	1 × 10^3^	Sheat (J/(kg⋅K))	1 × 10^3^
Thexp (1/K)	2 × 10^4^	Thexp (1/K)	2 × 10^4^

## Data Availability

The original contributions presented in this study are included in the article. Further inquiries can be directed to the corresponding authors.
